# Research on the Impact of Consumer Experience Satisfaction on Green Food Repurchase Intention

**DOI:** 10.3390/foods12244510

**Published:** 2023-12-18

**Authors:** Jing Wang, Shiwei Xu, Siyuan Zhang, Chen Sun, Linhai Wu

**Affiliations:** 1 College of Economics and Management, Shanghai Ocean University, Shanghai 201306, China; jwangocean@163.com (J.W.); 19862123951@163.com (S.Z.); chensun@shou.edu.cn (C.S.); 2 Institute of Food Safety Risk Management, Jiangnan University, Wuxi 214122, China; wyy@jiangnan.edu.cn

**Keywords:** green food, experience satisfaction, repurchase intention

## Abstract

With the continuous improvement in people’s living standards and the change in consumption concept, green food is favored by more and more consumers. Consumer repurchase behavior is a necessary condition to activate the market, expand the consumption scale and stabilize the continuous growth of the market. Repurchase intention is the most direct factor affecting consumers’ green food repurchase intention. Therefore, it is necessary to study consumers green food repurchase intentions. This study collects data from 303 consumer surveys on green food consumption to explore the impact of consumer satisfaction with consumption experience on green food repurchase intention and further explore the mechanisms and influence boundaries. The results show that (1) consumer experience satisfaction positively affects green food repurchase intention; (2) consumer experience satisfaction can improve consumers’ green food repurchase intention through consumer perceptions of social value, green self-efficacy and warm glow; (3) the higher the degree of consumer inertia, the stronger the influence of green self-efficacy and warm glow on consumers’ green food repurchase intention; and (4) the higher the degree of consumer subjective norms, the stronger the influence of consumer perceived social value, green self-efficacy and warm glow on the consumer’s green food repurchase intention. This study provides a new perspective and theoretical framework for promoting consumers’ green food repurchase intention, and it may have certain theoretical significance and practical impact on green food market growth, sustainable carrying of the ecological environment and high-quality development of agriculture.

## 1. Introduction

“Food is the basic of the people, security is the basic of the food”. Healthy and safe food is very important in daily life. With the continuous improvement in people’s living standards and the change in consumption concept, people’s food consumption concept has gradually changed from the initial low-level demand of “having enough to eat” to the high-level demand of “eating well”. Green food is a pollution-free, safe, high-quality and nutritious food produced by protecting the agricultural ecosystem and improving the quality of agricultural products and processed food to support the sustainable development of the national economy and society [1]. Compared with general food, green food is healthy, safe and pollution-free and is favored by more and more consumers. Green food manufacturers need to have loyal customers for their long-term development, and consumers’ intention to purchase green food is the most direct factor affecting the purchase behavior of green food [2]. According to the “Citizen’s Ecological and Environmental Behavior Survey Report 2020”, only about 29.3% of the respondents regularly buy green food. Therefore, it is necessary to study consumers’ intention to purchase green food again, which has realistic significance and far-reaching influence on the healthy growth of the green food market, the sustainable development of the ecological environment and the high-quality upgrade of agriculture.

The development of research on consumers’ green consumption behavior can be roughly divided into two stages. In the initial stage, scholars mainly analyzed which consumers are more inclined to green consumption by comparing the differences in demographic characteristics, such as gender, age, income, occupation and education level of consumers [3,4]. However, there are also studies that show that there is a weak correlation between these demographic characteristics and consumers’ green consumption behavior [5]. Subsequently, scholars’ research gradually turned to the influence of psychological factors, cognitive factors, external factors and other factors on consumers’ green consumption behavior [6,7,8]. Scholars introduced these deep-seated factors to study consumers’ green consumption behavior, which made the research results more explanatory [9]. When studying the green consumption behavior of consumers, most of the existing literature regards green consumption behavior as a single or initial behavior, and the consequences predicted by this model based on the assumption of a single behavior may be accidental, ignoring the consistency or repeatability of the behavior, which can no longer meet the current research and management needs [10]. In fact, there are obvious differences between green repurchase behavior and initial or single green consumption behavior. Therefore, the influencing factors and formation mechanisms of consumers’ green repurchase behavior need to be further explored [11].

Previous studies have shown that consumer experience satisfaction plays a key role in consumers’ green repurchase consumption behavior [12,13]. Green food consumption is a kind of consumption behavior. Although the existing research reveals that consumer experience satisfaction is the antecedent factor that affects consumers’ intention to purchase green food again [14], its mechanism and boundary of action need to be further studied. Therefore, this study intends to explore the impact of consumer satisfaction with their consumption experience on their intention to repurchase green food and further investigate the mediating mechanisms of consumer perceived social value, green self-efficacy and warm glow between consumer satisfaction with green food and their intention to repurchase green food, as well as the moderating mechanisms of consumer inertia and subjective norms. It aims to identify the transmission path and boundary conditions of consumer green repurchase intention and thus reveal the mechanism of consumer satisfaction with their consumption experience in relation to their intention to repurchase green food.

The rest of this study is arranged as follows: the second part is the theoretical basis and research hypothesis, which puts forward research hypotheses and constructs a research model by combining relevant theories; the third part is the research design, which explains the data source and research variables; the fourth part launches the empirical analysis; and finally, our research conclusions are discussed.

## 2. Theoretical Basis and Research Hypotheses

### 2.1. Consumer Experience Satisfaction (GreSat) and Green Food Repurchase Intention (RepurGre)

Consumer experience satisfaction comes from the theory of customer satisfaction, which is the consumer’s judgment on the degree to which the product (or service) itself or its characteristics meet their own needs [15]. Satisfaction is the subjective perception of consumers’ hearts, and subjective psychological feelings will greatly affect consumer repurchase intention and behavior [16]. From the perspective of consumer psychology and behavioral economics, consumers will expect quality and performance in a product before consuming it. If the perception of actual consumption exceeds the expectation, it will often lead to higher consumer experience satisfaction and a higher tendency to buy again. The opposite will lead to lower consumer experience satisfaction, and consumers may tend to buy similar products or substitutes from other brands when they make their next consumption [17]. In 1965, Cardozo introduced consumer experience satisfaction into the study of consumer repurchase intention for the first time and thought that consumer experience satisfaction was an important inducement to consumer repurchase intention [18]. Later, Bearden and Teel also confirmed that consumer experience satisfaction is an important determinant of consumer repurchases [19]. In their study of consumers’ consumption of green products, Ariffin et al. and Pahlevi et al. found that the satisfaction of the consumer is an important factor affecting consumers’ repurchase of green products [12,13]. Accordingly, this paper puts forward the following hypothesis:

**H1.**  *Consumer experience satisfaction has a positive impact on green food repurchase intention.*

### 2.2. The Mediating Role of Perceived Social Value (PerSocVa)

Perceived value is a subjective evaluation formed by consumers when they buy products or services [20]. Commodity trading needs to provide consumers with value, which is the basis for maintaining trading relations [21]. Perceived value is not only based on the function and attributes of the product but also derived from the consumer’s self-cognition. Based on the influence of green consumption on others and social interests, Koller and others put forward the concept of perceived social value, which refers to the value generated by green products by enhancing consumers’ social self-awareness; that is, if consumers think that their purchase behavior can be recognized by others, the perceived social value of the products will be enhanced [22]. Since then, Sweeney and Soutar believe that when consumers buy products, they consider the impression that buying behavior has on others [23]. Costa et al. believe that the perception of social value is very important to consumers’ food choices, and the social value of food consumption can be reflected in “what kind of food you eat symbolizes your social image” [24].

There are two different views on the relationship between perceived value and consumer satisfaction with consumption experience in academic circles. One is the satisfaction–value causal chain, with consumer experience satisfaction as the cause and perceived value as the result. The other is the value–satisfaction causal chain, with perceived value as the cause and consumer experience satisfaction as the result [25]. Kotler and Levy believe that perceived value is determined by the satisfaction of consumers’ consumption experiences [26]. In this study, consumption experience satisfaction is set as the antecedent variable to realize the transition from the first food consumption to the next consumption. Moreover, we think that, compared with the value cognition established before purchase, consumers will stimulate their understanding of product satisfaction in the product experience after purchase, which will produce a more comprehensive and profound perception of product value. Therefore, consumer satisfaction with the consumption experience will positively affect perceived value, and perceived value will continue to trigger consumer repurchase intentions and behaviors. Green food consumption is a kind of pro-environmental behavior that also makes consumers perceive its social value and further stimulates them to buy again. Accordingly, this paper puts forward the following hypothesis:

**H2.**  *Perceived social value plays an intermediary role in the influence of consumer experience satisfaction on green food repurchase intention.*

### 2.3. The Mediating Role of Green Self-Efficacy (GrSelEf)

Self-efficacy refers to the individual’s judgment and estimation of whether they have the ability to achieve their set goals [27]. Self-efficacy is always associated with a specific field. Green self-efficacy is a concept extended by integrating green environmental factors on the basis of the concept of self-efficacy, which refers to the individual’s evaluation of their ability to perform different activities to achieve green goals [28]. According to the existing research, green self-efficacy, as a kind of self-cognition, has a positive impact on green behavior [29]. In terms of green consumption, some scholars have found that green self-efficacy has a positive impact on green purchase intention [30]. In addition to the research on the mechanism by which green self-efficacy simply affects pro-environmental behavior, some scholars have also found that people are likely to gain knowledge and experience after implementing pro-environmental behavior once, thus guiding the next pro-environmental behavior [31]. It can be inferred that the green self-efficacy produced by consumers after the first consumption of green food can promote the next consumption of green food; that is, green self-efficacy positively affects green food repurchase intention.

Bandura found through extensive research that the formation of and change in self-efficacy are influenced by four kinds of information sources (direct experience, alternative learning, social environment persuasion and physical and mental state), which respectively convey certain efficacy information and affect people’s efficacy level, among which direct experience has the greatest influence on individual efficacy. For example, the experience of success or failure of behavior and personal experience from individuals have the greatest influence on self-efficacy. A successful experience can improve an individual’s sense of self-efficacy, while a failed experience will reduce an individual’s sense of self-efficacy [32]. This shows that the consumption experience of green food is the direct experience of consumers, and the satisfaction or dissatisfaction with this experience is similar to the success or failure of personal experiences, which will affect the production of green self-efficacy in consumers. Accordingly, this paper puts forward the following hypothesis:

**H3.**  *Green self-efficacy plays an intermediary role in the influence of consumer experience satisfaction on green food repurchase intention.*

### 2.4. The Mediating Effect of Warm Glow (WarmGlow)

Warm glow refers to the spiritual rather than material satisfaction and pleasure that individuals derive from impure altruism; it is impure altruism mixed with egoism rather than pure altruism [33,34,35]. Previous studies have shown that pro-environmental behavior is driven by warm glow [36,37,38], and warm glow is an important emotional intermediary to produce repeated pro-environmental behavior, which has also been confirmed in the field of neurology [39]. Studies have proved that the pro-environmental behavior caused by warm glow will make the reward area of the individual’s brain active, thus causing the individual to repeatedly implement the pro-environmental behavior. Pro-environmental behavior driven by warm glow can make individuals receive positive emotional encouragement and good social praise from this behavior [40,41]. Green consumption, as a kind of pro-environmental behavior, will also be strengthened by warm glow, which can positively affect consumer repurchase intention and sustainable green consumption behavior. Green food consumption can not only meet the basic functional needs of individuals for green food but also meet the social image needs and emotional needs of individuals for this behavior. After consumers have a sense of satisfaction in the consumption experience of green food, this sense of satisfaction can arouse consumers’ warm feelings, and it is easier for consumers to receive positive emotional resonance, thus stimulating the intention and behavior of repurchasing green food. Accordingly, this paper puts forward the following hypothesis:

**H4.**  *Warm glow plays an intermediary role in the influence of consumer experience satisfaction on green food repurchase intention.*

### 2.5. The Moderating Effect of Consumer Inertia (CusIner)

Inertia is consumers’ unconscious habitual purchase behavior [42]. Consumers make the same choice as the last consumption activity out of habit and to avoid consuming time and energy [43,44]. Carrasco et al. used panel data to observe whether consumers’ consumption behavior is inert and found that consumers do have inert behavior in food and service consumption [45]; that is, consumers often unconsciously buy goods they have chosen repeatedly [46] or have a tendency to continue to buy the same product [47,48]. Studies have confirmed that, even in situations of high conversion costs, consumers with high inertia will still choose the previous goods [42]. In addition, unless this consumption habit cannot be carried out as scheduled, consumers will tend to spend again on subsequent consumption due to inertia [49]. Consumers with high inertia, out of habit, and low intention to spend time and energy on shopping will repeat the purchase to avoid energy consumption as much as possible, while consumers with low inertia will choose whether to continue to buy the previous goods according to their purchasing motivation. Therefore, the higher the degree of consumers’ inertia, the stronger the influence of green self-efficacy and warm glow on consumers’ green food repurchase intention. Accordingly, this paper puts forward the following hypotheses:

**H5.**  *Consumer inertia plays a positive regulatory role in the relationship between perceived social value and green food repurchase intention.*

**H6.**  *Consumer inertia plays a positive role in the relationship between green self-efficacy and green food repurchase intention.*

**H7.**  *Consumer inertia plays a positive regulatory role in the relationship between warm glow and green food repurchase intention.*

### 2.6. The Moderating Effect of Subjective Norms (SubNorm)

The concept of subjective norms originated from rational behavior theory. Subjective norms refer to the influence of individuals or groups who have influence on individual behavior decisions on whether individuals take a specific behavior [50]. In the process of consumer decision-making, although consumers have initially decided to engage in some kind of purchase behavior, due to the demonstration effect of surrounding groups, sometimes there will still be involuntary behavior intentions, and then they will make different or even opposite decisions, which will eventually lead to behavioral changes [51]. Yang believes that Chinese people have a high tendency to obey social expectations and social orientation in their behavioral decision-making, which makes Chinese consumers attach great importance to social acceptance and external opinions and then leads them to adopt behaviors consistent with social norms when making decisions [52]. Therefore, subjective norms are a very important factor that affects the intention and behavior of Chinese consumers. Consumers with higher subjective norms will be more easily influenced by the consumption behavior of people around them and then decide their own consumption behavior. For consumers with low subjective norms, the consumption behavior of people around them has little influence on them, and consumers will not easily change their consumption behavior. There are also some scholars who try to use subjective norms as regulating variables to explain behavior. Li and others found that subjective norms regulated the relationship among altruism, self-efficacy, organizational support and knowledge sharing [53]. Therefore, the higher the degree of consumers’ subjective norms, the stronger the influence of consumers’ perceived social value, green self-efficacy and warm glow on consumers’ green food repurchase intention. Accordingly, this paper puts forward the following hypotheses:

**H8.**  *Subjective norms play a positive regulatory role in the relationship between perceived social value and green food repurchase intention.*

**H9.**  *Subjective norms play a positive role in the relationship between green self-efficacy and green food repurchase intention.*

**H10.**  *Subjective norms play a positive regulatory role in the relationship between warm glow and green food repurchase intention.*

**Figure 1:**
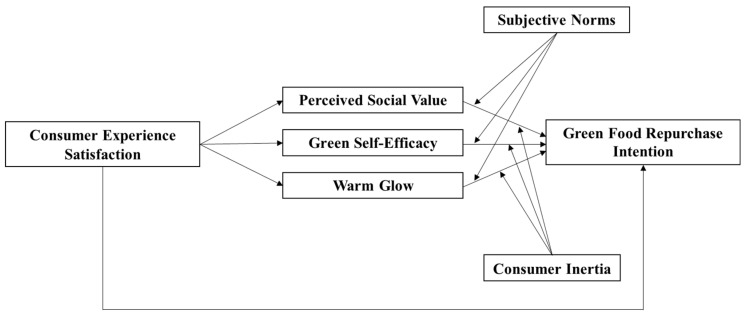
Research model framework diagram.

In summary, the framework diagram of the research model in this article is shown in Figure 1.

## 3. Research Design

### 3.1. Data Sources

The data in this paper come from online and offline consumer questionnaires. The offline questionnaire is mainly for a small-scale pre-survey to ensure the rationality of the questionnaire. The online questionnaire is mainly to expand the sample size and sample range. A total of 358 questionnaires were collected. After excluding “green food that has not been purchased” and incomplete and irregular samples, 303 valid questionnaires were finally obtained, and the effective rate of sample recovery was 84.6%. In order to ensure that the questions were reasonably set and the respondents understood clearly, we conducted a small-scale pre-investigation in Shanghai after the first draft of the questionnaire was completed. According to the feedback from the respondents, it was found that the selection of various topic indicators and the design of the questionnaire length were reasonable, but some words were too specialized, which led to a lack of clarity or ambiguity for the respondents. Accordingly, this study modified the expressions of items that caused difficulty or ambiguity and improved the rationality and scientific nature of the questionnaire, thus leading to the development of the final survey questionnaire. In terms of regional heterogeneity, we obtained some samples in the eastern, central and western provinces of mainland China. In terms of occupational heterogeneity, we paid attention to collecting data from groups of different occupational categories. When collecting data, we tried to randomly select subjects within each layer, so we think that the samples are representative. The basic characteristics of the sample are shown in Table 1.

### 3.2. Variable Selection

The variables selected in this paper include consumer experience satisfaction, green food repurchase intention, perceived social value, green self-efficacy, warm glow, consumer inertia and subjective norms. In order to ensure the reliability and effectiveness of the questionnaire measurement, the measurement items refer to the mature scale in the existing related research, and some items are reasonably modified and adjusted in combination with the characteristics of green food consumption and research topics. The questionnaire consists of three parts: The first part plays the role of screening. After reading the concept of green food and watching several pictures of products marked with “green food”, the filler needs to answer whether they have bought green food. Only by filling in “Yes” can the follow-up questions be answered. If they fill in “No”, the questionnaire will be answered directly, and the data filled in this part will be eliminated to ensure that the filler is a suitable survey object. The second part is the basic information of the respondents, including their gender, age, education level, monthly disposable income, professional nature and other information. The third part is composed of seven scales (consumer satisfaction with consumption experience, green food repurchase intention, perceived social value, green self-efficacy, warm glow, consumer inertia and subjective norms). They are measured by the Likert Level 5 Scale, which allows respondents to choose the level that suits their attitude; that is, the subjects score from “very disagree” to “very agree”, respectively. Details of the scale are are shown in Table 2.

### 3.3. Model Description

Referring to the existing literature [59,60,61,62,63], this study uses SPSS 22.0 statistical software and the multi-level linear regression method to explore whether consumer experience satisfaction will affect consumers’ green food repurchase intention, that is, to verify whether Hypothesis 1 is established. The mediating effect test method is used to further explore whether perceived social value, green self-efficacy and warmth effect play a mediating role in the influence of consumer experience satisfaction on green food repurchase intention, that is, to verify whether Hypotheses 2–4 are established. The moderating effect test method is used to explore whether consumer inertia and subjective norms play a moderating role in the relationship between perceived social value, green self-efficacy, warmth effect and green food repurchase intention, that is, to verify whether Hypotheses 5–10 are established.

## 4. Results

### 4.1. Data Reliability and Validity

Taking 303 valid questionnaires as research samples, this paper analyzes the reliability of the main latent variables using SPSS 22.0 software. As shown in Table 3, the Cronbach α coefficients of consumer experience satisfaction (GreSat), green food repurchase intention (RepurGre), perceived social value (PerSocVa), green self-efficacy (GrSelEf), warm glow (WarmGlow), subjective norm (SubNorm) and consumer inertia (CusIner) are 0.846, 0.856, 0.920, 0.887, 0.909, 0.902 and 0.813, respectively, which are all greater than the critical value of 0.7, indicating that the variable composition selected in this study has good reliability. Meanwhile, the average variance extracted (AVE) of each latent variable is greater than 0.5, and the combined reliability (CR) is greater than 0.7, indicating that each latent variable of the scale has good convergent validity.

As shown in Table 4, the results of confirmatory factor analysis show that the square root of the average variance extracted (AVE) of each latent variable is greater than the correlation coefficient between it and other latent variables. For example, the square root of the AVE of the latent variable of customer satisfaction with consumer experience (GreSat) is about 0.593. This value is greater than the correlation coefficient between consumer experience satisfaction (GreSat) and other latent variables. The results show good discriminant validity among the variables.

### 4.2. Variable Description Statistics and Pearson Correlation Coefficient

As shown in Table 5, there is a significant positive correlation between consumer experience satisfaction and green food repurchase intention (r = 0.637, *p* < 0.01). In addition, most variables have a good correlation relationship, and the Pearson correlation coefficient analysis results provide good evidence for the hypothesis test in the following text. Meanwhile, by comparing the mean and standard deviation values of each variable, it can be found that the degree of data dispersion is not high and the sample has good quality.

### 4.3. Regression Analysis

This section uses SPSS 22.0 to perform regression tests on the research hypotheses proposed earlier, and the results are shown in Table 6. Model (1) is the benchmark model with only control variables included, with an R-squared value of approximately 0.038. Model (2) added a key independent variable—consumer experience satisfaction (GreSat)—on the basis of the benchmark model, and the results showed that it had a significant positive effect on the dependent variable (r = 0.554, *p* < 0.01). The overall significance of the model was significant, and the R-squared value was significantly improved compared to the benchmark model. Original Hypothesis 1 was confirmed.

Meanwhile, we used the PROCESS v4.1 plugin to test the mediating effect of original Hypotheses 2–4. Model (3) tests the mediating effect of perceived social value (PerSocVa) on the impact of consumer experience satisfaction on green food repurchase intention. The indirect effect coefficient of perceived social value (PerSocVa) is 0.161, and the 95% confidence interval CI [0.102, 0.226] does not include a value of 0. In summary, perceived social value (PerSocVa) plays a significant mediating role in the positive relationship between consumer experience satisfaction (GreSat) and green food repurchase intention, and the data results support original Hypothesis 2. Model (4) shows that green self-efficacy (GrSelEf) has a positive impact on green food repurchase intention (r = 0.320, *p* < 0.01), while consumer experience satisfaction (GreSat) has a significantly positive impact on green food repurchase intention, and the coefficient is smaller than in model (2). At the same time, the indirect effect coefficient of green self-efficacy (GrSelEf) on green food repurchase intention is 0.127, with a 95% confidence interval of CI [0.072, 0.188], which does not include a value of 0. In summary, original Hypothesis 3 has been confirmed. Similarly, Model (5) tests the mediating effect of warm glow on the dependent variable. The indirect effect coefficient of warm glow on the dependent variable is 0.252, with a 95% confidence interval of CI [0.180, 0.329], which does not include a value of 0. Overall, original Hypothesis 4 is confirmed.

On the other hand, Table 7 reports the regression results of the moderating effect, where Model (1) shows that the interaction between consumer inertia and perceived social value did not reach statistical significance, meaning that consumer inertia did not regulate the positive impact of perceived social value on green food repurchase intention. Original Hypothesis 5 is not confirmed. The regression coefficient of the interaction term between consumer inertia and consumer green self-efficacy (GrSelEf) in model (2) is significantly positive (r = 0.149, *p* < 0.01), and in the comparison of the mean, below one standard deviation and above one standard deviation of the consumer inertia variable, it is found that the higher the value of the consumer inertia moderating variable, the more significant the influence of the independent variable on the dependent variable. In summary, consumer inertia has an enhanced moderating effect on the relationship between consumer green self-efficacy and green food repurchase intention, and original Hypothesis 6 is valid. The interaction term between consumer inertia and consumer warm glow in model (3) is significantly positive, and it is found that the higher the value of consumer inertia, the more significant the influence of the independent variable on the dependent variable is. In summary, the data results support original Hypothesis 7 by comparing the mean of the moderating variable and the values below and above one standard deviation. Similarly, models (4), (5) and (6) represent the test results for H8, H9 and H10, respectively, and the data results support these three original hypotheses.

## 5. Discussion and Conclusions

This study uses data from 303 consumer surveys on green food consumption to explore the impact of consumer experience satisfaction on green food repurchase intention and further investigate the mediating mechanisms of consumer perceived social value, green self-efficacy and warm glow between consumer experience satisfaction and green food repurchase intention, as well as the moderating mechanisms of consumer inertia and subjective norms. It aims to identify the transmission path and boundary conditions of consumer green food repurchase intention and thus reveal how the mechanism of consumer experience satisfaction impacts green food repurchase intention. Our research results are as follows:

Firstly, consumer experience satisfaction positively affects green food repurchase intention, which means that Hypothesis 1 is valid. This conclusion is consistent with the findings of Ariffin et al. [12] and Pahlevi et al. [13]. They believe that consumer experience satisfaction is an important factor that affects green food repurchase. Although existing research has revealed that consumer experience satisfaction is an important factor that affects consumers’ green food repurchases [14], there is a lack of research on its mechanism of action.

Secondly, perceived social value, green self-efficacy and warm glow play an intermediary role in the influence of consumer experience satisfaction on green food repurchase intention, which means that Hypotheses 2–4 are valid. Kotler et al.’s [26], Albert’s [32] and Andreoni’s [33] research respectively confirmed that consumer experience satisfaction will positively affect consumers’ perceived social value, green self-efficacy and warm glow, while Rizwan et al.’s [64], Thogersen et al.’s [31] and Sheng et al.’s [11] respectively confirmed that consumers’ perceived social value, green self-efficacy and warm glow will lead to repurchase intention. These scholars only expounded on the relationship between consumer experience satisfaction, consumer repurchase intention, consumers’ perceived social value, green self-efficacy and warm glow but did not link them to analyze their relationship. This study not only proves that consumer experience satisfaction can have a direct impact on green food repurchase intention but also proves that the effect of consumer experience satisfaction on green food repurchase intention is achieved through the mediation of perceived social value, green self-efficacy and warm glow.

Thirdly, consumer inertia has no significant moderating effect on the relationship between perceived social value and green food repurchase intention, while consumer inertia has a positive moderating effect on the relationship between green self-efficacy, warm glow and green food repurchase intention; that is, Hypothesis 5 is not valid, and Hypotheses 6 and 7 are valid. Consumers’ subjective norms play a positive regulatory role in the relationship between perceived social value, green self-efficacy, warm glow and green food repurchase intention; that is, Hypotheses 8–10 are valid. Consumer inertia and subjective norms have a moderating effect, and this conclusion is the same as that of Tsai et al. [49] and Li et al. [53], respectively. From the perspective of consumer inertia adjustment, for consumers with high inertia, consumers are out of habit and have low intention to spend time and energy on shopping, and they will repurchase to avoid energy consumption as much as possible, while consumers with low inertia will choose whether to continue to buy the previous goods according to their purchasing motives. Due to the regulatory role of subjective norms, consumers with higher subjective norms will be more easily influenced by the consumption behavior of people around them and then decide their own consumption behavior. For consumers with low subjective norms, the consumption behavior of people around them has little influence on them, and consumers will not easily change their consumption behavior. In the moderating role, the moderating role of consumer inertia in the relationship between perceived social value and green food repurchase intention is not significant. The possible reason for this situation lies in the particularity of green food, that is, perceived social value refers to the value generated by green products by enhancing consumers’ social self-awareness, which is very important for consumers’ food choice [24]. Commodity trading needs to provide value for consumers, and this value is the basis for maintaining the trading relationship [21]; that is, once consumers form perceived social value in the process of green food consumption, they will not be easily influenced by other factors and change their purchase decisions. Therefore, consumers’ perception of social value positively affects the repurchase intention of green food, and it is not easily affected by the inertia of consumers.

The marginal contribution of this study may be as follows: (1) Theoretical implications: Firstly, previous studies have paid little attention to consumers’ green food repurchase behavior, and more attention has been paid to the first or single green food consumption behavior. This study reveals the influencing factors and formation mechanisms of consumers’ green food repurchase intention, which enriches the research on consumers’ green consumption behavior. Secondly, previous studies only generally studied the direct effect of consumer satisfaction with consumption experience on consumers’ green food resale, while further research on the intermediary mechanism and regulation mechanism is not perfect. This study systematically explored the functional path and boundary conditions between consumer satisfaction with consumption experience and consumers’ green food repurchase, which is of great significance for enriching the theoretical framework of the effect of consumer satisfaction with consumption experience on green food resale intention. (2) Practical implications: First, enterprises should pay attention to the driving effect of consumer experience satisfaction on consumers’ green repurchase intention when carrying out green marketing. Consumers’ consumption experience satisfaction is consumers’ recognition of green food. Enterprises should ensure the quality of green food and convey the functional or tangible benefits brought by green food consumption behavior to consumers so that consumers can be satisfied with their first consumption experience, thereby improving their self-efficacy and encouraging them to continue to buy green food. Secondly, enterprises should emphasize the social value benefits generated by their green food consumption behavior, such as environmental performance, reputation image, etc., and convey the warmth signal of green food consumption behavior to consumers, emphasizing the emotional benefits brought by green consumption behavior to consumers, so as to promote the continuous occurrence of consumers’ repeated purchase behavior.

The limitations of this paper are as follows: (1) In the selection of research variables, the influencing factors and mechanisms of consumers’ repurchase intention and behavior are more complicated, and most of the current research is exploratory research. This study mainly focuses on how the mechanism of consumers’ green food consumption experience satisfaction impacts green food repurchase intention without considering other factors. Therefore, in the future, more relationship elements should be explored to understand the impact of consumers’ green food repurchase intention and behavior more deeply and comprehensively. (2) In terms of data collection, the sample size of the survey data may have a certain impact on our research results. The amount of data in this study is limited, and subsequent research can broaden the sample size of the data.

## Figures and Tables

**Table 1:** Basic characteristics of samples.

Indicators	Categories	Frequency	Proportion (%)	Indicators	Categories	Frequency	Proportion (%)
Gender	Men	117	38.6	Monthly disposable income	Below USD 420	142	46.9
	Women	186	61.4		USD 420–820	83	27.4
Age	Under 18	3	1		USD 820–1220	30	9.9
	18–30	158	52.1		USD 1220–1620	20	6.6
	31–40	42	13.9		Over USD 1620	28	9.2
	41–50	53	17.5	Education level	Below senior high school	14	4.6
	Over 50	47	15.5		Senior high school or technical secondary school	23	7.6
Professional Nature	Civil servants	7	2.3		Undergraduate or junior college	223	73.6
	Enterprise employees	67	22.1		Graduate students or above	43	14.2
	Public institution employees	52	17.2	Regions	East	183	60.4
	Students	134	44.2		Central	100	33.0
	Self-employed employees	14	4.6		West	15	4.9
	Others	29	9.6		Others	5	1.7

**Table 2:** Variable definition and description.

Variable	Index	Code	Item	Source
Independent Variable	Consumer experience satisfaction (GreSat)	GS1	The environmental value generated by consuming green food exceeds my expectations.	Wu [54] (2019)
		GS2	I sincerely feel satisfied with the consumption experience of green food.	
		GS3	Based on all my food consumption experiences, I believe that purchasing green food is a wise choice.	
		GS4	I think green food consumption can contribute to environmental protection and sustainable development.	
Dependent Variable	Green food repurchase intention (RepurGre)	RG1	I will continue to purchase green food in the future.	Liu [55] (2021)
		RG2	If I happen to see green food online or offline in supermarkets, I will choose to purchase it.	
		RG3	I am willing to try more green food in the future.	
Mediator Variable	Perceived social value (PerSocVa)	SV1	Whenever I hear someone praising me for eating green food, I feel very happy.	
		SV2	If my peers could notice that I consume green food, I would be very happy.	
		SV3	If I had the opportunity to tell others that I eat green food, I would feel good.	
		SV4	Eating green food gives me the opportunity to showcase my lifestyle to others. If I consume green food, it will leave a positive impression on others.	
	Green self-efficacy (GrSelEf)	SE1	I think I have the ability to help achieve environmental goals.	Du [56] (2022) Chen [57] (2001)
		SE2	I think I can effectively fulfill my environmental mission.	
		SE3	I think I have the ability to effectively handle environmental issues.	
		SE4	I think we can find creative ways to solve environmental problems.	
	Warm glow (WarmGlow)	WG1	Buying green food is beneficial for environmental protection, which can bring me a happy mood and a sense of personal achievement.	Hartmann [35] (2017)
		WG2	I am willing to contribute to human welfare and the quality of the natural environment, such as purchasing green food.	
		WG3	I am happy to do some good deeds for our Earth home, such as purchasing green food to reduce environmental pollution.	
		WG4	I am very satisfied that purchasing green food can give back to society and the ecological environment.	
Moderator Variable	Consumer inertia (CusIner)	CI1	Unless I am extremely disappointed with the green food I have purchased, I will not replace it.	Anderson [35] (2003)
		CI2	I found it difficult to replace the previously purchased green food.	
		CI3	For my consumption, replacing green food that I have previously purchased would waste a lot of time, energy, and money.	
	Subjective norms (SubNorm)	SN1	My family believes that I should buy green food instead of non-green food.	Joshi [58] (2021)
		SN2	Most of the people I respect will buy green food instead of non-green food.	
		SN3	The people I respect believe that I should buy green food.	

**Table 3:** Reliability and convergence validity of variables.

Variable	Number	Cronbach α	AVE	CR
GreSat	4	0.846	0.593	0.851
RepurGre	3	0.856	0.669	0.858
PerSocVa	4	0.920	0.744	0.921
GrSelEf	4	0.887	0.666	0.888
WarmGlow	4	0.909	0.718	0.910
SubNorm	3	0.902	0.759	0.904
CusIner	3	0.813	0.607	0.820

**Table 4:** Differential validity test for latent variables.

	GreSat	RepurGre	PerSocVa	GrSelEf	WarmGlow	SubNorm	CusIner
AVE	0.593	0.668	0.744	0.666	0.718	0.759	0.607
SQR(AVE)	0.770	0.818	0.862	0.816	0.847	0.871	0.779
GreSat							
RepurGre	0.746						
PerSocVa	0.518	0.67					
GrSelEf	0.435	0.633	0.677				
WarmGlow	0.615	0.811	0.761	0.738			

**Table 5:** Variable description statistics and Pearson correlation coefficient (N = 303).

	GreSat	RepurGre	PerSocVa	GrSelEf	WarmGlow	SubNorm	CusIner
GreSat	1						
RepurGre	0.637 ***	1					
PerSocVa	0.464 ***	0.610 ***	1				
GrSelEf	0.396 ***	0.567 ***	0.619 ***	1			
WarmGlow	0.415 ***	0.560 ***	0.695 ***	0.669 ***	1		
SubNorm	0.428 ***	0.540 ***	0.585 ***	0.624 ***	0.672 ***	1	
CusIner	0.360 ***	0.389 ***	0.446 ***	0.497 ***	0.504 ***	0.530 ***	1
Mean	3.923	4.025	3.907	3.837	4.111	3.682	3.760
S D	0.702	0.625	0.758	0.717	0.668	0.796	0.722

Note: *** *p* < 0.01.

**Table 6:** Mediation effect test (N = 303).

	(1)	(2)	(3)	(4)	(5)
VARIABLES	Repurchase	Repurchase	Repurchase	Repurchase	Repurchase
WarmGlow					0.487 ***
					(0.042)
GrSelEf				0.320 ***	
				(0.038)	
PerSocVa			0.329 ***		
			(0.037)		
GreSat		0.554 ***	0.394 ***	0.427 ***	0.302 ***
		(0.040)	(0.040)	(0.039)	(0.040)
Age	0.054	0.036	−0.002	0.022	0.020
	(0.037)	(0.029)	(0.026)	(0.026)	(0.024)
Educ	0.174 ***	0.113 **	0.078 *	0.093 **	0.051
	(0.062)	(0.049)	(0.044)	(0.044)	(0.041)
Gender	0.036	−0.004	−0.006	0.014	−0.069
	(0.078)	(0.061)	(0.054)	(0.055)	(0.051)
MonSalary	0.017	0.001	0.013	−0.002	0.015
	(0.054)	(0.027)	(0.024)	(0.024)	(0.022)
Constant	3.260 ***	1.414	0.953 ***	0.763	0.709
	(0.250)	(0.235)	(0.216)	(0.225)	(0.205)
R-squared	0.038	0.419	0.540	0.531	0.599
F-value	2.969 **	42.822 ***	57.879 ***	55.875 ***	73.610 ***

Note: Standard errors in parentheses; *** *p* < 0.01, ** *p* < 0.05, * *p* < 0.1. The same notation applies later.

**Table 7:** Regulatory effect test (N = 303).

	(1)	(2)	(3)	(4)	(5)	(6)
VARIABLES	Repurchase	Repurchase	Repurchase	Repurchase	Repurchase	Repurchase
SubNorm						0.067 **
WarmGlow						(0.030)
SubNorm					0.061 **	
GrSelEf					(0.031)	
SubNorm				0.074 **		
PerSocVa				(0.033)		
CusIner			0.093 **			
WarmGlow			(0.043)			
CusIner		0.149 ***				
GrSelEf		(0.047)				
CusIner	0.173					
PerSocVa	(0.046)					
SubNorm				−0.456	0.016	−0.143
				(0.128)	(0.128)	(0.132)
CusIner	0.064	−0.455	−0.369			
	(0.194)	(0.191)	(0.190)			
WarmGlow			0.314 *			0.348 ***
			(0.161)			(0.110)
GrSelEf		−0.129			0.105	
		(0.178)			(0.116)	
PerSocVa	0.376 **			0.091		
	(0.174)			(0.125)		
Control	yes	yes	yes	yes	yes	yes
R-squared	0.407	0.377	0.529	0.452	0.407	0.545
F-value	28.954 ***	25.551 ***	47.241 ***	34.800 ***	28.908 ***	50.59 ***

Note: Standard errors in parentheses; *** *p* < 0.01, ** *p* < 0.05, * *p* < 0.1.

## Data Availability

The data presented in this study are available on request from the corresponding author. The data are not publicly available due to restrictions eg privacy or ethical.
